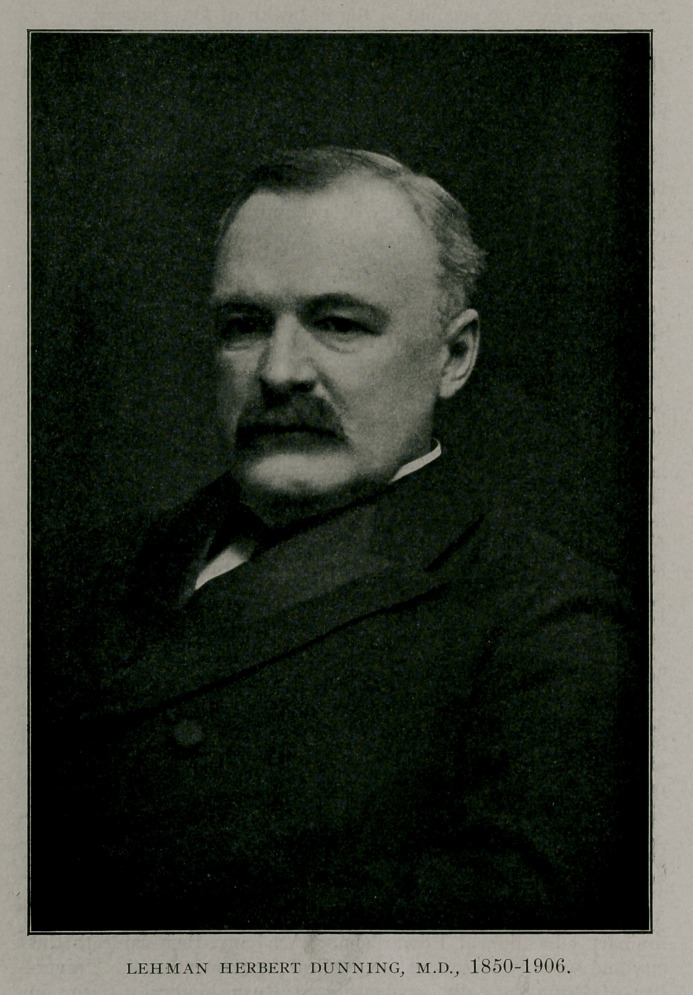# Dr. Lehman Herbert Dunning

**Published:** 1906-01

**Authors:** 


### OBITUARY.

Dr. Lehman Herbert Dunning, of Indianapolis, Ind., died at his home in that city Thursday morning, January 4, 1906, aged 55 years. The news of his sudden death came as a shock to his many friends all over the country. He appeared in usual health during the last day of his life, kept his usual office hours, lectured at the Indiana Medical College, and performed other professional duties. He retired about 11 o'clock, and was seized two hours later with acute precordial pain. A physician was called who remained until the patient seemed relieved, after which Dr. Dunning himself insisted that he should return to his home. At 3 o'clock he was sleeping peacefully and the family, wife, son and daughter, went to their rooms and to bed. At 5 o'clock Mrs. Dunning observed from the door of his room that he was quiet and supposed that he was asleep. At 6 his daughter called to him because of a telephone message, but on receiving no response, went to his bedside and discovered he was dead. And this is the brief story of the last illness and death of this great surgeon!

Lehman Herbert Dunning was born at Edwardsburg, Mich., April 12, 1850, his father being a substantial farmer. His grandfather, Dr. Isaac D. Dunning, was a leading practitioner at Aurora, Erie Co., N. Y., for thirty years, but removed to Michigan in 1836. The subject of this memoir received his preliminary education at Edwardsburg Academy, then came to this city and spent two years at the medical department of the University of Buffalo, finally graduating at Rush Medical College in 1872. Dr. Dunning began medical practice at Troy, Mich., went to South Bend, in 1878, and to Indianapolis in 1889. He took

LEHMAN HERBERT DUNNING, M.D., 1850-1906.

post-graduate instruction in this country and in Europe, going abroad three several times for research and study. He was invited by the faculty of Indiana Medical College to take the chair made vacant by the death of Dr. L. B. Harvey, and thus became professor of Gynecology and Abdominal surgery in that institution, where he lectured less than twenty-four hours before his death, as before stated.

Dr. Dunning held membership and filled office in the local, state, and national medical societies. He was chairman of the section on gynecology of the American Medical Association in 1903 and was president of the American Association of Obstetricians and Gynecologists during the same year.

Dr. Dunning was a man of genial temper, sunny presence, and a pleasing address. He was able and justly distinguished in his profession; a contributor to the literature of medicine; and a teacher of forcefulness who readily obtained the confidence and respect of his medical classes. He will be missed by the profession in this country and in Europe, by a large circle of immediate patients and friends, but most of all by a deeply attached family, consisting of a wife, two sons, and a daughter, who receive the sympathy of all who knew their distinguished husband and father.

## Figures and Tables

**Figure f1:**